# A Rapidly Progressive Bullous Eruption in a Toddler

**DOI:** 10.7759/cureus.101419

**Published:** 2026-01-13

**Authors:** Ayesha Khalid, Sundus Ghouri, J. Sebastian Proano

**Affiliations:** 1 Pediatrics, Marshall University Joan C. Edwards School of Medicine, Huntington, USA; 2 Medicine, Fatima Memorial College of Medicine and Dentistry, Lahore, PAK; 3 Pediatric Intensive Care, Marshall University Joan C. Edwards School of Medicine, Huntington, USA

**Keywords:** bullous eruption, crown of jewels sign, dapsone therapy, direct immunofluorescence, pediatric autoimmune blistering disease, pediatric dermatology, subepidermal blister, vesiculobullous disorders

## Abstract

Linear IgA bullous dermatosis (LABD) is a rare autoimmune blistering disorder characterized by subepidermal vesiculobullous eruptions and a variable clinical presentation. In pediatric patients, LABD may present as rapidly progressive and widespread disease, often mimicking other severe blistering conditions and complicating early diagnosis. We report a case of a toddler with a two-week history of a progressive bullous eruption involving the extremities, lower abdomen, perioral region, and genitals, with sparing of the oral mucosa. Examination revealed a combination of flaccid hemorrhagic bullae and tense vesicles, with a negative Nikolsky sign. The extent and morphology of the eruption prompted evaluation for multiple serious blistering disorders. Histopathologic examination with direct immunofluorescence demonstrated linear IgA deposition along the basement membrane zone, confirming the diagnosis of LABD. The patient was treated with systemic corticosteroids followed by dapsone, resulting in marked clinical improvement and near-complete resolution. This case emphasizes the importance of recognizing atypical and rapidly evolving presentations of LABD and highlights the role of early biopsy and timely immunomodulatory therapy in achieving favorable outcomes.

## Introduction

Linear IgA bullous dermatosis (LABD), also referred to as chronic bullous disease of childhood, is an uncommon autoimmune blistering condition characterized by subepidermal vesiculobullous eruptions and linear IgA deposition along the basement membrane zone [1,2]. Pediatric presentations are heterogeneous, ranging from localized annular lesions to rapidly progressive, widespread eruptions that may resemble other severe blistering disorders [3,4]. A distinctive clinical feature, described as the “crowns of jewels” or “string of pearls” sign, is characterized by multiple tense vesicles or bullae arranged in a ring, with new lesions forming at the periphery of an erythematous plaque, creating a beaded, annular outline and is reported in approximately 60% of pediatric cases, occurring more frequently in children than in adults [3,5]. Given its rarity and significant clinical overlap with life-threatening dermatologic conditions, prompt recognition of characteristic morphologic features is critical to facilitate accurate diagnosis and timely management [4,5]. We describe a toddler with a rapidly progressive bullous eruption that underscores the diagnostic importance of this classic pattern and the role of early immunomodulatory therapy.

## Case presentation

**Figure 1:**
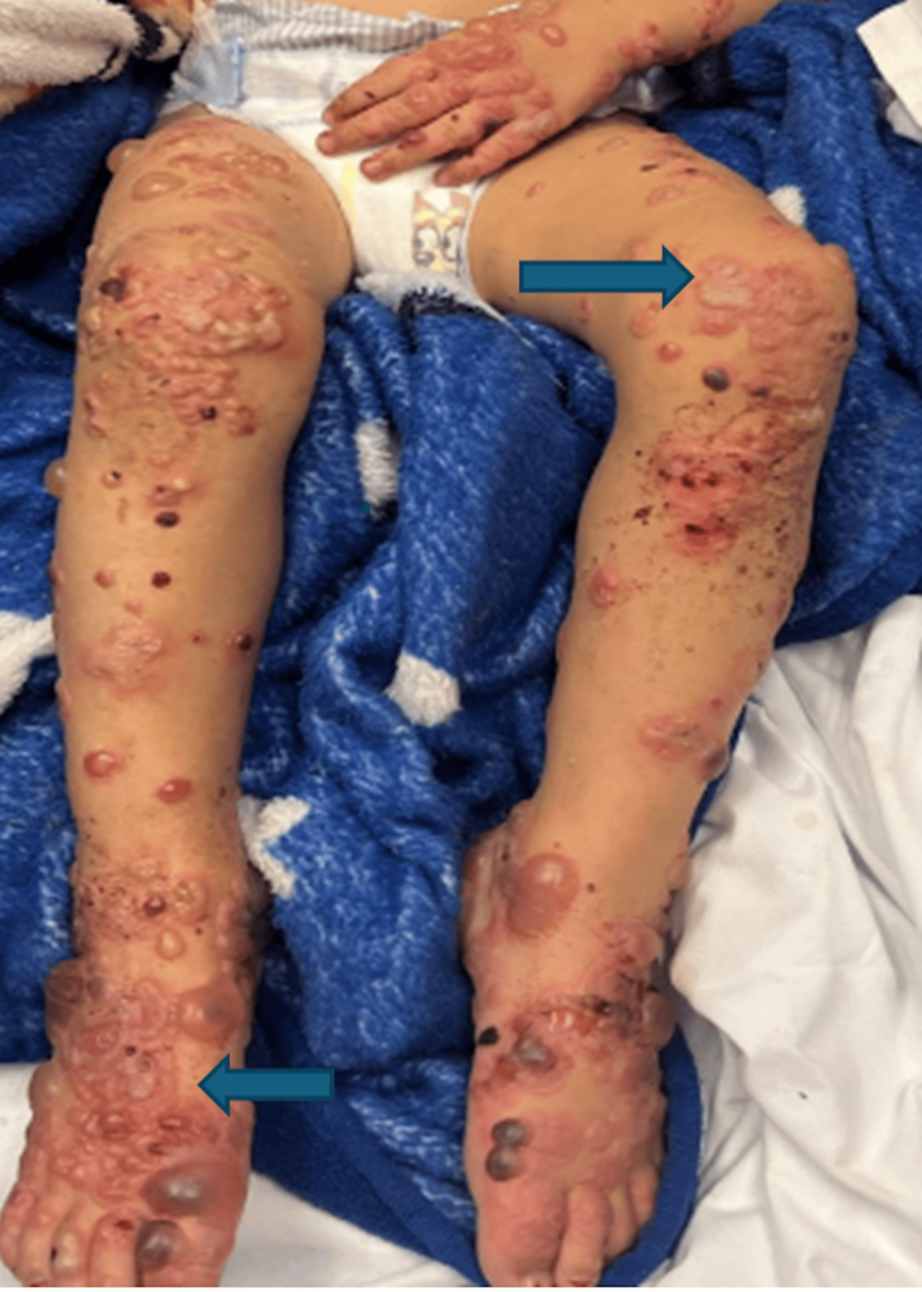
Annular tense vesicles forming the classic “crown of jewels” pattern

**Figure 2:**
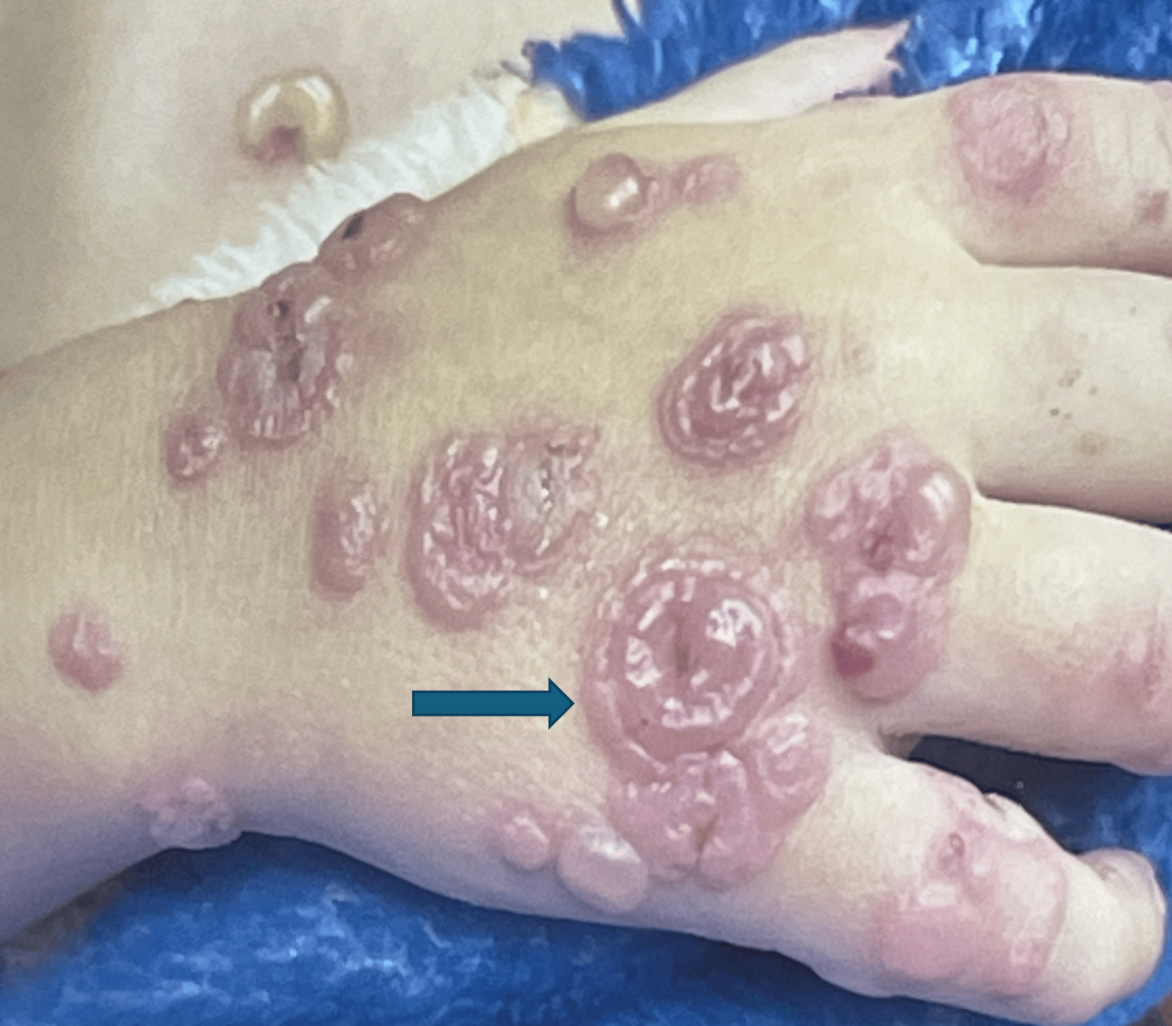
Crowns of jewels appearance

A two-year-old male toddler presented with a rapidly progressive, widespread vesiculobullous eruption involving the extremities, lower abdomen, perioral region, and genitals while sparing the mucosal surfaces (Figure 1). He was fully immunized for age, with no significant past medical history, no recent medication use, and no known family history of dermatologic disease. The child appeared ill with decreased oral intake and signs of dehydration, though he remained hemodynamically stable at the time of presentation. On physical examination, the bullae and vesicles were mixed in type: some were flaccid and hemorrhagic while others were tense and demonstrated the classic “crown of jewels” pattern, with new vesicles forming in a concentric arrangement around older lesions (Figure 2). The Nikolsky sign was negative. Given the rapid progression, widespread involvement, and mixed lesion morphology, the differential diagnosis included autoimmune vesiculobullous disorders such as linear IgA dermatosis, bullous pemphigoid, and other pediatric blistering diseases.

Given the extent and severity of cutaneous involvement, the differential diagnosis encompassed several vesiculobullous conditions, including autoimmune disorders such as LABD, bullous pemphigoid, Stevens-Johnson syndrome, and toxic epidermal necrolysis. A coordinated care approach was promptly undertaken with consultation from pediatric dermatology and wound care teams.

Wound care included daily cleansing with hypochlorous acid solution, application of topical bacitracin to individual lesions, and occlusive coverage using petrolatum-impregnated gauze. Additional laboratory evaluation, including glucose-6-phosphate dehydrogenase (G6PD) testing, was obtained in anticipation of potential systemic therapy.

**Figure 3:**
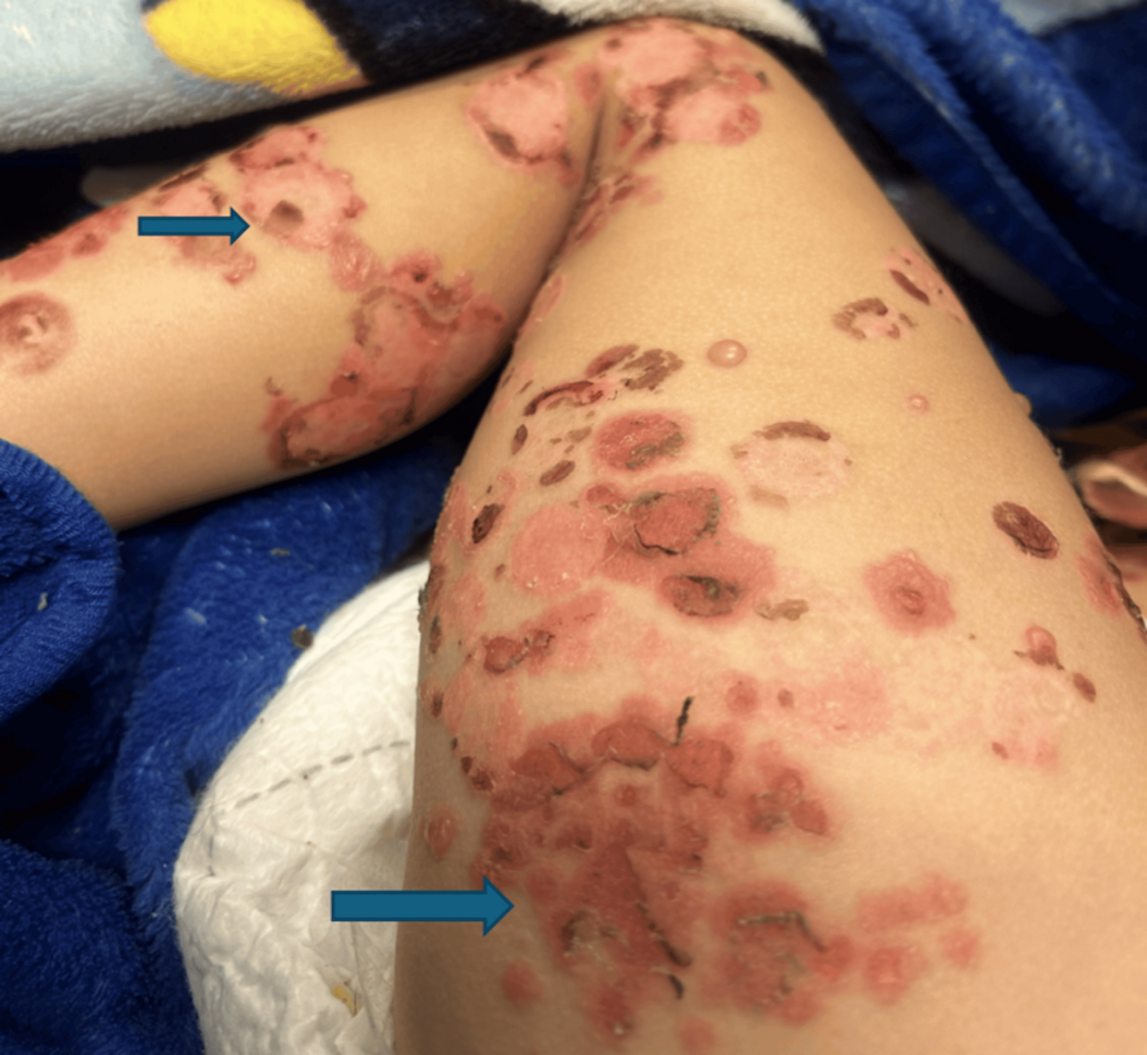
Clinical improvement following corticosteroid therapy in linear IgA bullous dermatosis

By hospital day 2, the morphology of the lesions evolved, with several bullae rupturing and becoming flaccid with hemorrhagic bases, while new vesicles continued to develop. By day 4, ongoing lesion formation prompted initiation of dapsone following confirmation of normal G6PD activity, and lesional skin biopsies were obtained. Mild clinical improvement was observed after dapsone initiation, and a short course of systemic corticosteroids was subsequently added. Following corticosteroid therapy, the patient demonstrated progressive improvement, with cessation of new lesion formation and crusting of existing lesions (Figure 3).

**Figure 4:**
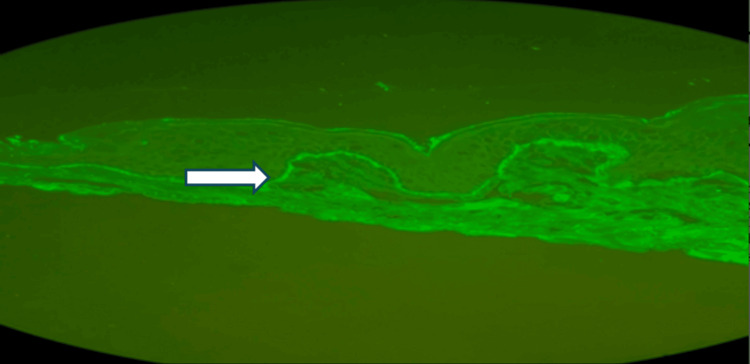
Linear IgA deposition along the BM BM: basement membrane

Histopathologic evaluation with direct immunofluorescence later confirmed LABD (Figure 4). With sustained clinical improvement, the patient was discharged on dapsone at 2 mg/kg/day with outpatient dermatology follow-up.

This case underscores the diagnostic complexity of autoimmune blistering diseases in young children presenting with rapidly progressive bullous eruptions. Recognition of evolving lesion morphology, timely biopsy, and stepwise initiation of immunomodulatory therapy were central to disease control and clinical recovery.

## Discussion

Linear IgA dermatosis, also known as chronic bullous dermatosis of childhood, is an autoimmune blistering disorder in which IgA autoantibodies target components of the basement membrane zone, resulting in subepidermal vesiculobullous eruptions [1-3]. The typical age of onset is approximately five years; however, younger children may present with more extensive or rapidly progressive disease. Commonly affected areas include the limbs, trunk, head, buttocks, and perioral region [3,4]. Perioral involvement in the absence of mucosal disease remains an important diagnostic clue.

This case was diagnostically challenging due to rapid progression, extensive body surface area involvement, and the coexistence of both flaccid and tense bullae features that overlap with several acute pediatric blistering disorders, including bullous pemphigoid, Stevens-Johnson syndrome, and toxic epidermal necrolysis [5,6]. Nevertheless, the characteristic "crown of jewels" or "string of pearls" configuration, marked by peripheral vesicles forming around older lesions, remains a distinguishing feature of linear IgA dermatosis and is reported more frequently in pediatric populations [3,4,7]. A negative Nikolsky sign further supported this diagnosis.

Definitive diagnosis requires a lesional skin biopsy with direct immunofluorescence demonstrating linear IgA deposition along the basement membrane zone [7,8]. Because biopsy confirmation is not immediate, and disease progression may be rapid, early therapeutic intervention is often necessary to limit further blister formation and skin involvement.

The key features that distinguish this condition from other childhood bullous disorders are the classic hallmark configuration of lesions, a negative Nikolsky sign, the lack of mucosal involvement, and the absence of systemic toxicity, which together help differentiate it from staphylococcal scalded skin syndrome, bullous pemphigoid, Stevens-Johnson syndrome, and toxic epidermal necrolysis.

Dapsone remains the first-line therapy for linear IgA dermatosis and is effective through inhibition of neutrophil-mediated inflammation [1,4,7]. In cases of rapidly evolving or widespread disease, a short course of systemic corticosteroids may be added to achieve faster disease control and suppress new lesion formation [1,2,4]. Evidence guiding pediatric treatment strategies remains limited; therefore, management is largely informed by clinical experience, retrospective cohorts, and expert consensus [4,6]. The marked clinical improvement observed after initiation of dapsone with adjunctive corticosteroids in this patient is consistent with outcomes reported in other pediatric series [1,3,4,9]. This case underscores the importance of recognizing characteristic morphologic features of linear IgA dermatosis and initiating timely immunomodulatory therapy to prevent disease progression and promote recovery.

## Conclusions

This case highlights the diagnostic and therapeutic challenges of LABD in young children presenting with rapidly progressive bullous eruptions. The clinical overlap with other severe blistering disorders can delay recognition and treatment. In this patient, an early skin biopsy was essential for confirming the diagnosis, and prompt initiation of dapsone with adjunctive corticosteroids resulted in significant clinical improvement.
